# Polymorphic Crystallization Behavior of a Poly(butylene adipate) Midblock within a Poly(L-lactide-butylene adipate-L-lactide) Triblock Copolymer

**DOI:** 10.3390/polym14224902

**Published:** 2022-11-13

**Authors:** Lei Hua, Xiaodong Wang

**Affiliations:** 1 Institute of Polymer Science and Engineering, TongJi Zhejiang College, Jiaxing 314051, China; 2 Jiaxing Key Laboratory of High-Performance and Functional Materials in Civil and Environmental Engineering, Tongji Zhejiang College, Jiaxing 314051, China

**Keywords:** biodegradable aliphatic, triblock copolymers, polymorphic crystal

## Abstract

New biodegradable aliphatic PLLA-PBA-PLLA copolymers with soft poly(butylene adipate) (PBA) and hard poly(l-lactide) (PLLA) building blocks were synthesized via ring-opening polymerization (ROP). Proton nuclear magnetic resonance (^1^HNMR) was utilized to confirm the volume fraction of PBA (f_PBA_) within PLLA-PBA-PLLA. It was found that a PBA midblock (PBA-mid) within PLLA-PBA-PLLA-s (PLLA-PBA-PLLA triblock copolymer with a short PLLA block length) might display lamellar domain structure. However, PBA-mid within PLLA-PBA-PLLA-l (PLLA-PBA-PLLA triblock copolymer with a long PLLA block length) might locate itself as a nanoscale cylindrical domain surrounded by a PLLA continuous phase. Polymorphic crystals of PBA-mid within the PLLA-PBA-PLLA copolymers were formed after melt crystallization at the given temperatures, which were studied by differential scanning calorimetry (DSC) and wide-angle X-ray diffraction (WAXD) analysis. According to the WAXD and DSC analyses, it was interesting to find that the α-type crystal of PBA-mid was favorable to develop in the lower temperature region regardless of the state (crystallization or amorphous) of the PLLA component. Additionally, when the PLLA component was held in its amorphous state, it was easier for PBA-mid within the PLLA-PBA-PLLA copolymers to transform from the metastable β-form crystal to the stable α-form crystal. Furthermore, polarized optical microscopy (POM) photos provided direct evidence of the polymorphic crystals of PBA-mid within PLLA-PBA-PLLAs.

## 1. Introduction

Poly(butylene adipate) (PBA) is a type of widely studied, biodegradable, aliphatic polyester with lower glass transition (T_g_) at −54 °C [1]. The melting temperature (T_m_) and crystallization temperature (T_c_) of PBA are 50~60 and ∼30 °C, respectively [2]. The α-, β-form crystals and a mixture of both crystal structures of polymorphic crystals of PBA have been identified under different thermal conditions [3]. The α-form crystal is characterized as a monoclinic unit cell with the dimensions of a = 6.73 Å, b = 7.94 Å, and c = 14.20 Å (fiber axis). While for the β form, PBA chains are packed in an orthorhombic unit cell with the dimensions of a = 5.06 Å, b = 7.35 Å, and c = 14.67 Å (fiber axis) [1]. The temperature-induced polymorphic crystals of PBA have been further studied by DSC and WAXD analyses [1]. Gan et al. [1] reported that the β-form crystal grew more favorably in a temperature region of below about 29 °C, and the α-form crystal mainly formed above 31 °C. In the temperature range from 29 to 31 °C, a mixture including β- and α-form crystals developed. In addition, β and α crystals of PBA show the corresponding melting behavior, which allows us to correlate the crystal structure with the crystal size, melting point, and crystallization temperature, and subsequently, to demonstrate the mechanisms of polymorphic crystal growth and transformation. Generally, the crystal grown at a higher temperature has higher thermodynamic stability. Therefore, it is rational to regard the α crystal of PBA as a more stable phase compared to the β crystal [4,5]. Gan et al. [6] also reported that films with an α crystal structure displayed a faster biodegradation rate than that of films with a β crystal structure, although the α crystal is a thermodynamically stable phase with a larger crystal size.

Recently, considerable reports have been published on the study of poly(l-lactide) (PLLA), the most attractive and useful class of biodegradable polyesters, which possesses many desirable properties, such as nontoxicity, low immunogenicity, and good biocompatibility, for being applied in the biomedical and pharmaceutical fields [7,8,9]. It is also well-known that PLLA shows a melting point (T_m_) of ∼190 °C and a crystallization temperature (T_c_) of ∼120 °C [10]. However, the applications of PLLA to commercial products are limited principally by its brittleness. This weakness might be improved if a polymer with a lower glass transition (T_g_) temperature is employed to prepare blends or copolymers with PLLA [11]. The compounding of PLLA with other polymers has been employed to improve its physical properties; it has been blended with thermoplastic starch [12], poly(ethylene oxide) (PEO) [13], poly(ε-caprolactone) (PCL) [14], poly(3-hydroxybutyrate) (PHB) [15], etc. [16,17]. Although the blending offers a good way to strengthen and toughen brittle or stiff polymers by incorporating a soft or elastomeric second component, the system of the blending exhibits strong phase separation [18], and incompatible and poor mechanical properties [19]. In order to share the merits of biodegradable PBA and PLLA in their physical properties, it seems better to propose a new block copolymer bearing hard PLLA and soft PBA building blocks.

The so-called “ABA” triblock copolymers have two identical end “A” blocks covalently bonded to the middle “B” block. The bulk properties of symmetric ABA triblock copolymers have been studied extensively, both theoretically and experimentally [20,21]. It is reported that ABA triblock copolymers provide attractive applications, because they can assemble into structures with slightly smaller characteristic dimensions to those of AB diblock copolymers, and ABA triblock copolymers also exhibit a slightly narrower interfacial width in their weak segregation region than AB diblock copolymers [22,23]. Furthermore, a distinguished difference between triblock and diblock copolymers is that the central B block can form bridges between two different A domains within the ABA triblock copolymers, and bridging conformations in ABA triblock copolymers are believed to significantly improve their bulk mechanical properties [24]. Therefore, it seems relevant to prepare a new PLLA-PBA-PLLA triblock copolymer in this present research. It is well-known that crystallization conditions significantly affect crystalline structure, which is closely related to the mechanical and physical properties of crystals [25,26]. In the case of double crystalline block copolymers, the situation can be even more complicated because the state of one block component may manipulate the crystallization and morphology of the other block component. Therefore, the temperature-dependent polymorphic crystallization behavior of PBA-mid within PLLA-PBA-PLLA triblock copolymers under the different states (crystallization or amorphous) of the PLLA component, arouse our considerable interest.

Nurkhamidah and Woo [27] reported that the polymorphic crystals of PBA in PLLA/PBA blends showed the same temperature-dependent behavior as that of neat PBA under the crystallization condition of the PLLA component. The same issue is proposed for PLLA-PBA-PLLA triblock copolymers: How does the state of the PLLA component affect the polymorphic crystals of PBA-mid within PLLA-PBA-PLLA triblock copolymers under the different thermal programs? Whether or not the microdomain morphology of the PLLA-PBA-PLLA triblock copolymer in melts plays a significant role in controlling the polymorphic phenomena of melt crystallization in PBA-mid at given temperatures will be discussed thoroughly in this study. Furthermore, it is more important that the polymorphism of PBA-mid is firstly investigated within the semicrystalline/semicrystalline PLLA-PBA-PLLA triblock copolymer. This investigation of the polymorphic crystallization behavior of ABA triblock copolymers could establish a model system to control and regulate the structure of polymorphic polymers in semicrystalline/semicrystalline triblock copolymers.

In this study, PLLA-PBA-PLLA copolymers with soft PBA and hard PLLA building blocks were synthesized via ROP, and the number average (M_n_), and the mass average molecular weight (M_w_) of the PLLA-PBA-PLLA copolymers could be confirmed by gel permeation chromatography (GPC) and ^1^HNMR. The temperature-dependent polymorphic crystallization behavior of PBA-mid within the PLLA-PBA-PLLA triblock copolymers under two states (crystallization or amorphous) of the PLLA blocks was investigated by DSC and WAXD. The phase transition of PBA-mid by annealing within the PLLA-PBA-PLLA triblock copolymer was also investigated by WAXD. Finally, POM photos provided direct evidence of the polymorphic crystals of PBA-mid within the PLLA-PBA-PLLA copolymers.

## 2. Materials, Instruments, and Methods

### 2.1. Materials

The chemicals 1,4-butanediol, adipate acid, and Sn(Oct)_2_ were purchased from Shanghai Aladdin Industrial Corporation and used as received. L-lactide was purchased from Sigma-Aldrich Corporation and recrystallized from ethyl acetate 3 times before use. PLLA was synthesized by the ROP of L-lactide and precipitated in ethanol 3 times before use. The molecular weight of PLLA is summarized in Table 1. Bifunctional hydroxyl-capped PBA macroinitiator was prepared via a two-step procedure including esterification and subsequent polycondensation. First, 0.5 mol of adipate acid and 0.6 mol of 1,4-butanediol were charged into a 300-mL, three-necked, round-bottom flask equipped with a mechanical stirrer, under a nitrogen atmosphere. The esterification reaction was carried out at 190 °C and nitrogen flow was performed for a period of 4.0 h with continuous removal of the released water. Then, the condensation polymerization was held for 6 h at 190 °C. The viscous liquid product was cooled to room temperature under the nitrogen flow. Finally, the solid mass was dissolved in chloroform and precipitated with an excess amount of cold dry methanol. The precipitate was collected by filtration and extensively washed with copious amounts of cold methanol, and then dried under reduced pressure at 40 °C for 72 h. The M_n_ and M_w_ of bifunctional hydroxyl-capped PBA macroinitiator prepared are recorded in Table 1. On the basis of the prepared bifunctional hydroxyl-capped PBA macroinitiator, PLLA-PBA-PLLA triblock copolymers were further synthesized via the ROP of an L-lactide monomer with SnOct_2_ as the catalyst. A three-necked 100-mL flask was flamed and in turn charged with 5 g of the above prepared PBA, then, a SnOct_2_ toluene solution and a predetermined amount of L-lactide monomer in sequence. The reaction system was also purged with nitrogen and elevated to 120 °C in an oil bath, and further held for 24 h. As a result, the product was attained, further dissolved in chloroform, and precipitated by cold methanol. The precipitate was filtered and washed with an abundance of methanol 3 times and finally dried in a vacuum oven at room temperature. The obtained products were recorded as PLLA-PBA-PLLA-s and PLLA-PBA-PLLA-l, respectively, according to the length of PLLA block within the PLLA-PBA-PLLA copolymers, as shown in Table 1.

### 2.2. Instruments

Proton Nuclear Magnetic Resonance (^1^HNMR). ^1^HNMR spectra were measured at room temperature in a CDCl_3_ solution on a spectrometer (AvanceII 600 MHz, Bruker Corp., Fällanden, Switzerland) after 30 pulses, with 3.7 s-pulse repetition time, 32 K data points, and 256 FID accumulations.

Gel Permeation Chromatography (GPC). A Waters apparatus (1515-2414, Waters Corp., Milford, MA, USA), equipped with a refractive index detector, and Styragel HR columns packed with 5-µm particles were adopted for GPC analysis. Chloroform was used as the eluent at a flow rate of 1 mL/min and the sample concentration was 1 mg/mL. Polystyrene standards of low polydispersity were used to construct a calibration curve. The number average (M_n_) and mass average molecular weight (M_w_) could be obtained from the GPC data.

Differential Scanning Calorimetry (DSC). The thermal behavior of the samples was measured by DSC (Discovery DSC 250, TA Corp., Santa Fe Springs, CA, USA), with the thermal analysis station, under a nitrogen atmosphere. The temperature and heat flow at different heating rates were calibrated using an indium standard with nitrogen purging. The sample (5~8 mg) was weighted and sealed into an aluminum pan. For the isothermal crystallization, the sample was melted at 200 °C for 3 min to remove the thermal history. Then, it was quenched quickly at a rate of 100 °C/min to the desired crystallization temperature. Two thermal programs were applied in this research.

Wide-Angle X-ray Diffraction (WAXD). There were two thermal methods used in this experiment. The first thermal method entailed the samples being sandwiched between two iron plates with a thickness of 2 mm and pressed for 3 min at 200 °C under 5 MPa on a hot press. Then, the pressed sample films were quickly thrown into a water bath preset to the desired temperature. After the completion of isothermal crystallization, the films were measured at room temperature by WAXD. The second thermal method also involved the samples being sandwiched between two iron plates with a thickness of 2 mm and pressed for 3 min at 200 °C under 5 MPa on a hot press. The pressed films were quickly put into a vacuum oven at 90 °C for 40 min. The samples were then thrown into a water bath preset to the desired temperature as soon as possible. After the completion of the isothermal crystallization process, the films were measured at room temperature by WAXD. WAXD patterns were recorded on X-ray diffractometer (SmartLab, Rigaku Corp., Tokyo, Japan) with Ni-filtered CuKa radiation (λ = 0.1542 nm), worked at 40 kv and 200 mA. WAXD patterns were collected in the range of Bragg angles of 5~50 °C at a scanning rate of 1 °C/min.

Polarized Optical Microscopy (POM). POM observation was performed using microscopy (BX90, Olympus Corp., Tokyo, Japan) equipped with a digital camera. The sample was sandwiched between two glass slides and melted at 200 °C for 3 min on a hot stage. During melting, the sample was pressed slightly to form a thin film with a thickness of 0.1 mm. Then, it was quickly transferred to another hot stage preset to the desired temperature. The photographs were recorded after the completion of crystallization.

### 2.3. Methods

**Figure 1:**
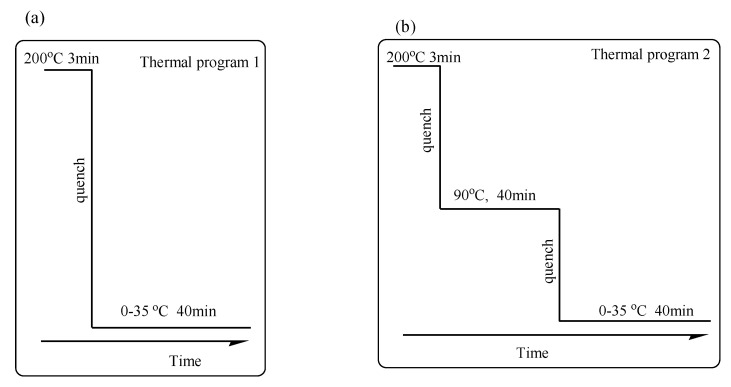
(**a**) Thermal program 1 and (**b**) thermal program 2 applied in DSC and WAXD analyses.

Two thermal programs were chosen to make sure the temperature depended on the polymorphic crystallization behavior of PBA-mid within the triblock copolymers. Figure 1a represents thermal program 1 and Figure 1b represents thermal program 2, which were utilized in DSC and WAXD analyses. In the thermal program 1, all samples were heated at 200 °C and held for 3 min, then quenched at a maximum rate to the melt crystallization temperature ranging from 0 to 35 °C of PBA. Because the T_g_ of PLLA (T_g,PLLA_) is located above the melt crystallization temperature of PBA, PLLA was held as the rigid amorphous phase in the thermal program 1. In this case, it is considered that a semicrystalline/semicrystalline block copolymer turns into a semicrystalline/amorphous one. Meanwhile in the thermal program 2, all samples were also firstly melted at 200 °C and held for 3 min. Then, they were quenched at a maximum rate to 90 °C and held for 40 min until the crystallization process of PLLA was relatively saturated, and then quenched to the given isothermal crystallization temperature for the crystallization process of PBA at a maximum rate. In the thermal program 1, quenching the temperature to T < T_g,PLLA_ leads to the amorphous state of the PLLA component, resulting in the transition from the fully amorphous melt state to the semicrystalline/amorphous two-phase state. Meanwhile, in the thermal program 2, decreasing the temperature to T = T_c,PLLA_ results in the transition from the fully amorphous melt state to the semicrystalline/semicrystalline two-phase state in sequence.

## 3. Results

### 3.1. Chemical Properties and Compositions

**Figure 2:**
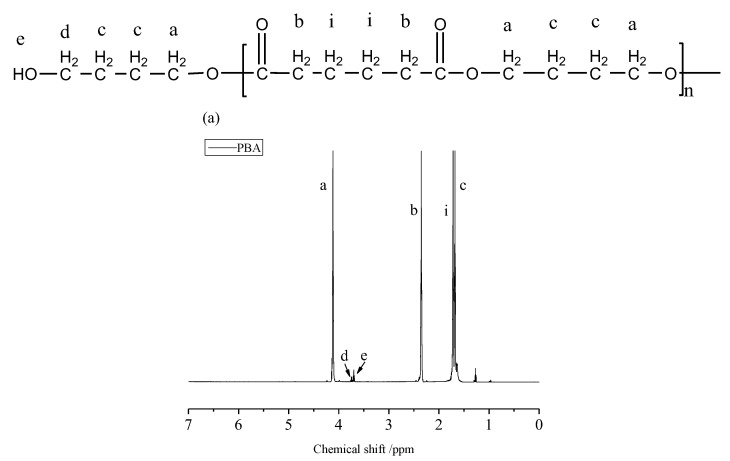

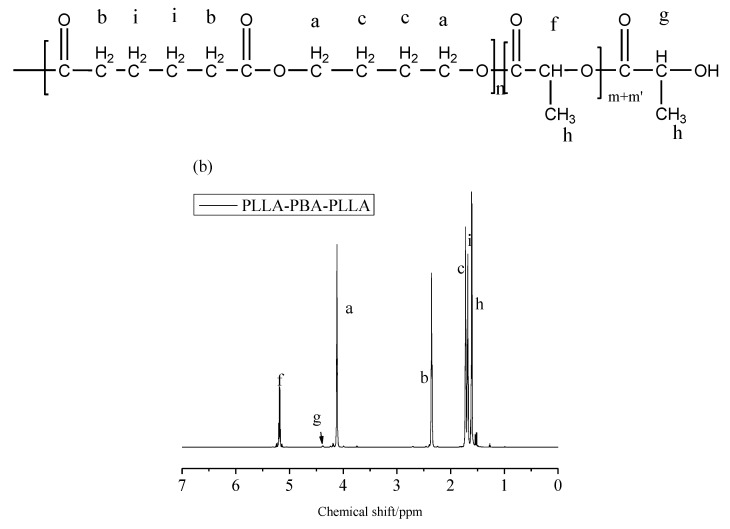
^1^HNMR spectra of (**a**) PBA and (**b**) PLLA-PBA-PLLA copolymers.

To synthesize the new biodegradable aliphatic PLLA-PBA-PLLA triblock copolymers, bifunctional hydroxyl-capped PBA macroinitiators should firstly be prepared. Commonly, low molecular weight is an inherent characteristic for aliphatic polyesters prepared by melt polycondensation due to the presence of competitive reactions between the polycondensation and thermal degradation that will concurrently occur [28]. Meanwhile, the aliphatic polyester end groups can be controlled by adjusting the initial feeding molar ratio of diol to diacid. With an excessive amount of 1, 4-butanediol, it can be expected that PBA macromolecular chains will predominantly possess hydroxyl end groups, although there may still exist small amounts of carboxyl end groups stemming from the accompanying degradation reactions [28]. Figure 2 shows the ^1^HNMR spectrum of the synthesized PBA macroinitiator with the corresponding assigned proton resonance signals. The proton resonance signals occurring at “d” and “e” can be reasonably assigned to the methylene proton and hydroxyl proton of HO-CH_2_CH_2_CH_2_CH_2_-. This experimental evidence proves that the synthesized linear PBA chains are terminated by hydroxyl functional end groups. Consequently, the number average molecular weight, denoted as Mn_NMR_ (shown in Table 1), could be calculated from the intensity ratio of the -OCH_2_CH_2_CH_2_CH_2_- methylene proton signal and HOCH_2_CH_2_CH_2_CH_2_- methylene proton signal occurring at 4.05 (a) and 3.65 (d) ppm, respectively.

As to the syntheses of PLLA-PBA-PLLA triblock copolymers, the ROP of L-lactide monomers was carried out in bulk with a prepared PBA prepolymer and SnOct_2_ as the catalyst under 120 °C. It has been reported that less or no transesterification reactions occur under such reaction conditions [29]. Figure 2b shows a typical ^1^HNMR spectrum of the copolymerization product. A new signal at δ = 4.35 ppm, attributed to the HO-CH- methine proton, is present [30]. This result clearly demonstrates that the PBA-derived hydroxyl group initiates the ROP of L-lactide and the copolymer chains are terminated by L-lactide-derived hydroxyl groups. Based on the ^1^HNMR spectra, the molar ratios of -OCH(CH_3_)CO-OCH(CH_3_)CO-(LA) to butylene adipate (BA) repeating units were determined from the corresponding intensities of the LA unit methine proton signals at 5.15 ppm and BA unit methylene proton signals at 4.05 ppm. The Mn_NMR_ values of the copolymers (shown in Table 1) were calculated from the sum of the Mn_NMR_ value of the PBA prepolymer and the Mn_NMR_ of the corresponding PLLA within the copolymers, determined from the intensity ratios of the OCH methine proton signals at δ = 5.15 ppm and HO-CH terminal methine proton signals at δ = 4.35 ppm. Clearly, the Mn_NMR_ values increased along with increased content of “LA ”repeating units in the copolymers. The GPC data are also summarized in Table 1. It was found that the GPC data agreed well with the results from the ^1^HNMR analysis. The volume fraction of PBA (f_PBA_) and PLLA (f_PLLA_) in the PLLA-PBA-PLLA copolymer was estimated from the result of the ^1^HNMR, using the homopolymer melt densities, ρ_PBA_ = 0.826 g/mL and ρ_PLLA_ = 1.154 g/mL [31].

### 3.2. Nonisothermal Crystallization Analysis by DSC

**Figure 3:**
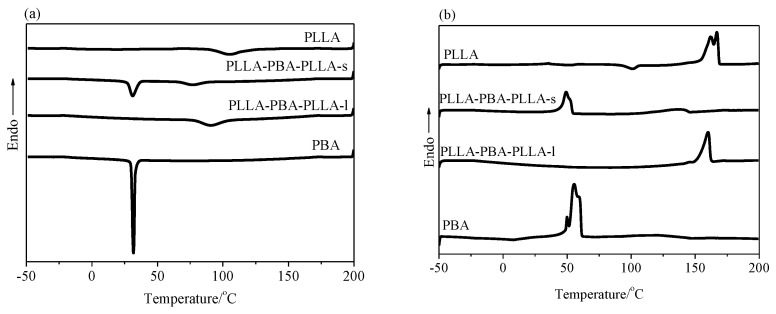
(**a**) DSC cooling curves at a scanning rate of 10 °C/min and (**b**) heating curves of PLLA, PBA, PLLA-PBA-PLLA-s, and PLLA-PBA-PLLA-l.

The thermal properties of PBA, PLLA, PLLA-PBA-PLLA-s, and PLLA-PBA-PLLA-l copolymers were characterized by means of DSC. Figure 3 shows the typical DSC curves of PLLA-PBA-PLLA triblock copolymers. All samples were subjected to a cooling and heating run. Figure 3a represents the cooling curves and Figure 3b shows the second heating curves of PBA, PLLA, PLLA-PBA-PLLA-s, and PLLA-PBA-PLLA-l. The results of the crystallization and melting temperatures of these samples are also summarized in Table 1. According to Figure 3a,b, crystallization and melting peaks in the low temperature region are related to PBA-mid, and those in the high temperature region, attributed to PLLA blocks. T_c,PLLA_ and T_m,PLLA_ are markedly higher than those of PBA. During the cooling process, the crystallization process of PLLA blocks has finished before reaching T_c,PBA_. It can clearly be observed that the T_c,PAB_, T_c,PLLA_, T_m,PBA_, and T_m,PLLA_, are seriously dependent on the f_PBA_ within the PLLA-PBA-PLLA copolymers. Because the f_PBA_ in PLLA-PBA-PLLA-s is about 68%, PBA-mid shows a slightly wide crystallization peak at 31.1 °C. However, for PLLA-PBA-PLLA-l with f_PBA_ = 35%, it is difficult to clearly observe the crystallization peak of PBA-mid, which might suggest that the excess amount of PLLA blocks confine the segment’s mobility and impede the crystallization behavior of PBA-mid dramatically during the fast cooling process.

### 3.3. Crystalline Structure of Neat PBA and PBA-mid Analyzed by WAXD and DSC

#### 3.3.1. Crystalline Structure of Neat PBA and PBA-mid Analyzed by WAXD

**Figure 4:**
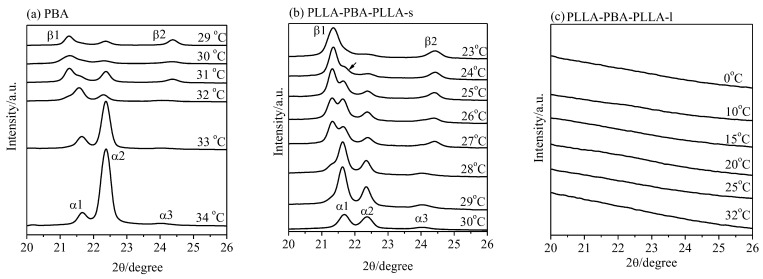
WAXD patterns of (**a**) PBA, (**b**) PLLA-PBA-PLLA-s, and (**c**) PLLA-PBA-PLLA-l after melt crystallization at different temperatures under the thermal program 1.

It is notable that the PBA crystal structure is very sensitive to the temperature and cooling process from melting to given crystallization temperature. The structural change of PBA-mid during the melt crystallization at various crystallization temperatures (T_c,PBA_) could be investigated from these diffraction patterns. WAXD analysis could provide a direct and precise method, rather than DSC analysis, to demonstrate the temperature region at which the α-, β-form crystals and transformation process of the α-, β-form crystals develop. For clarity, only the WAXD peaks of the PBA-mid are shown in Figure 4.

The pure β crystal of PBA appearing at T_c,PBA_ ≤ 29 °C, and the α crystal developing at T_c,PBA_ ≥ 34 °C of the neat PBA are shown in Figure 4a. At _c,PBA_ = 29~31 °C, a mixture of the α- and β-forms developed. The pure α form developed at 30 °C, which is lower than the upper critical formation temperature of the neat PBA (~34 °C). It was clearly found that the pure β-form crystal appears at 23 °C for PLLA-PBA-PLLA-s, which is much lower than the critical formation temperature of the neat PBA (~29 °C), as shown in Figure 4b. The crystal structure, including a mixture of α- and β-form crystals, appears after melt crystallization in the temperature range of 24~29 °C. However, no diffraction peak assigned to PBA-mid for PLLA-PBA-PLLA-l can be observed even when melt temperature deceased to T_c,PBA_ = 0 °C, as shown in Figure 4c. As a result, the temperatures at which the α crystal and the mixture of α- β-form crystals of PBA-mid form could shift to the lower temperature region under the thermal program 1.

**Figure 5:**
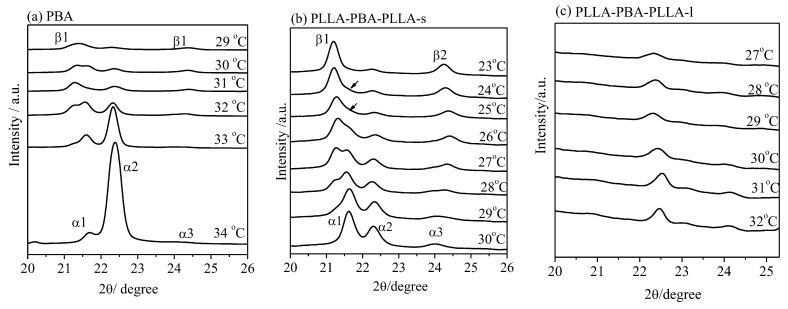
WAXD patterns of (**a**) PBA, (**b**) PLLA-PBA-PLLA-s, and (**c**) PLLA-PBA-PLLA-l after melt crystallization at different temperatures under the thermal program 2.

For PLLA-PBA-PLLA-s, it is interesting to identify that the crystal structure, including a mixture of α- and β-form crystals, appears after melt crystallization at 24 °C ≤ T_c,PBA_ ≤ 29 °C under the thermal program 1, as shown in Figure 5b. Moreover, the α-form crystal of PBA-mid within PLLA-PBA-PLLA-s also appears entirely at T_c,PBA_ = 30 °C, greatly lower than that for the neat PBA (34 °C), while the β-form crystals of PBA-mid appear entirely at T_c,PBA_ = 23 °C. According to Figure 5c, although the crystal of PBA-mid within PLLA-PBA-PLLA-l could be found, it is difficult to distinguish the α- and β-form crystals clearly due to the imperfect crystal of PBA-mid confined by PLLA blocks. Under the thermal program 2, it is also observed that the temperatures at which the α crystal form and the mixture of α-, β-form crystals of PBA-mid form are located in the lower temperature region.

#### 3.3.2. Crystalline Structure of Neat PBA and PBA-mid Analyzed by DSC

**Figure 6:**
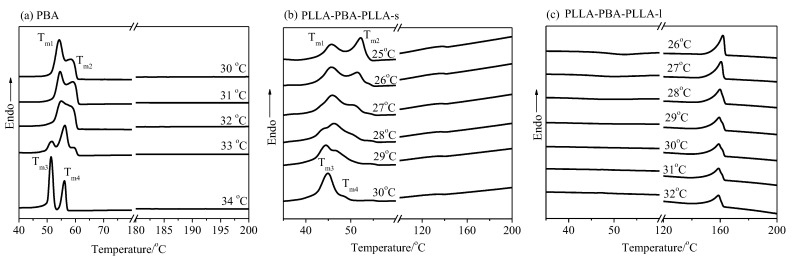
Melting curves of (**a**) PBA, (**b**) PLLA-PBA-PLLA-s, and (**c**) PLLA-PBA-PLLA-l after melt crystallization at a given temperature, and then heating directly from this given temperature at a heating rate of 10 °C/min under the thermal program 1.

In this work, DSC analysis was also employed to confirm the evidence of the polymorphic crystallization behavior of PBA-mid within PLLA-PBA-PLLA copolymers as compared with the neat PBA. Figure 6 shows the melting curves of the PBA, PLLA-PBA-PLLA-s, and PLLA-PBA-PLLA-l after melt crystallization at the given temperatures, under the thermal program 1. According to Figure 6a, except for the triple melting peaks that are observed at 32~33 °C, double melting peaks of the neat PBA are found in different crystallization temperature ranges. At the lower crystallization temperature of 30 °C, two melting peaks of T_m1_ and T_m2_ (β-form crystal) of the neat PBA are detected, and two new melting peaks T_m3_ and T_m4_ (α-form crystal) develop at T_c,PBA_ = 34 °C. The characteristic melting peaks of PBA-mid within PLLA-PBA-PLLA-s shown in Figure 6b could be found clearly. It is observed that the melting peaks of T_m1_ and T_m2_ (β-form crystal) occurring at T_c,PBA_ ≤ 25 °C decreased by 5 °C, compared to those of the neat PBA. On the other hand, the melting peaks of T_m3_ and T_m4_ (α-form crystal) occurred at T_c,PBA_ ≥ 30 °C. The T_c,PBA_ for the α-form crystal (30 °C) decreased by 4 °C, compared to that of the neat PBA. Furthermore, due to the transformation step of the α- and β-form crystals, the triple melting peaks appear at 26 °C ≤ T_c,PBA_ ≤ 29 °C, during the heating process. However, the melting peaks of PBA-mid could not be observed in PLLA-PBA-PLLA-l (See Figure 6c).

**Figure 7:**
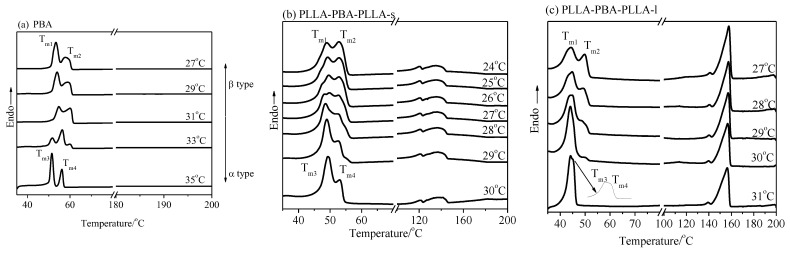
Melting curves of (**a**) PBA, (**b**) PLLA-PBA-PLLA-s, and (**c**) PLLA-PBA-PLLA-l after melt crystallization at a given temperature, and then heating directly from this given temperature at a heating rate of 10 °C/min under the thermal program 2.

Figure 7 shows the melting curves of the neat PBA, PLLA-PBA-PLLA-s, and PLLA-PBA-PLLA-l after melt crystallization at the given temperature under the thermal program 2. In this case, the PLLA phase is firstly crystallized at T_c,PLLA_ = 90 °C for 40 min until saturation. For PLLA-PBA-PLLA-s, the melting peaks of T_m3_ and T_m4_ could easily be found due to the α-form crystal appearance at T_c,PBA_ ≥ 30 °C, while the melting peaks of T_m1_ and T_m2_ could be found due to the β-form crystal occurrence at T_c,PBA_ ≤ 24 °C, shown in Figure 7b. Moreover, the melting peaks of T_m3_ and T_m4_ were found due to the α-form crystal appearance at T_c,PBA_ ≥ 31 °C, while the melting peaks of T_m1_ and T_m2_ were found due to the β-form crystal occurrence at T_c,PBA_ ≤ 27 °C for PLLA-PBA-PLLA-l, shown in Figure 7c.

#### 3.3.3. Compared Results of WAXD and DSC

Table 2 summarizes the melt crystallization temperatures of the samples, at which the α-, β-form crystals appear under the thermal 1 and 2 programs based on WAXD and DSC analyses. According to the results of WAXD, it is observed that the α-form crystal of the neat PBA and PBA-mid within the PLLA-PBA-PLLA-s appears at 34 and 30 °C, respectively, under the thermal program 1. That is, the crystallization temperature of the α-form crystal for the PBA-mid within PLLA-PBA-PLLA-s decreased by 4 °C compared to the neat PBA. It can be concluded that the α-form crystal of PBA-mid favorably grows at a lower temperature within the PLLA-PBA-PLLA copolymers. A similar phenomenon could be observed in the thermal program 2, the melt crystallization temperature at which the α-, β-form crystals appeared decreasing greatly for PBA-mid within the PLLA-PBA-PLLA copolymers, as compared with the neat PBA. Furthermore, the polymorphic crystallization behavior of PBA-mid within PLLA-PBA-PLLA-l under the thermal program 2 could be observed, but not in the thermal program 1. Table 2 summarizes the melt crystallization temperatures of the samples, at which α-, β-form crystals appear under the thermal programs 1 and 2, measured by DSC. A depression was clearly observed in the values of the melting temperatures, T_m1_, T_m2_, T_m3_, and T_m4_ of PBA-mid within the PLLA-PBA-PLLA copolymers under the thermal program 2. However, the melting peaks of PBA-mid within PLLA-PBA-PLLA-l under the thermal program 1 could not be detected by DSC.

It also needs to be mentioned that the DSC results are not the same as the WAXD results discussed above, which is attributable to the difference in the cooling rate between the WAXD and DSC measurements. For the WAXD measurement, the sample was directly thrown into a water bath preset to the desired T_c,PBA_. However, for the DSC measurement, it needed a short time to reach the T_c,PBA_ even at a high cooling rate of 100 °C/min.

**Figure 8:**
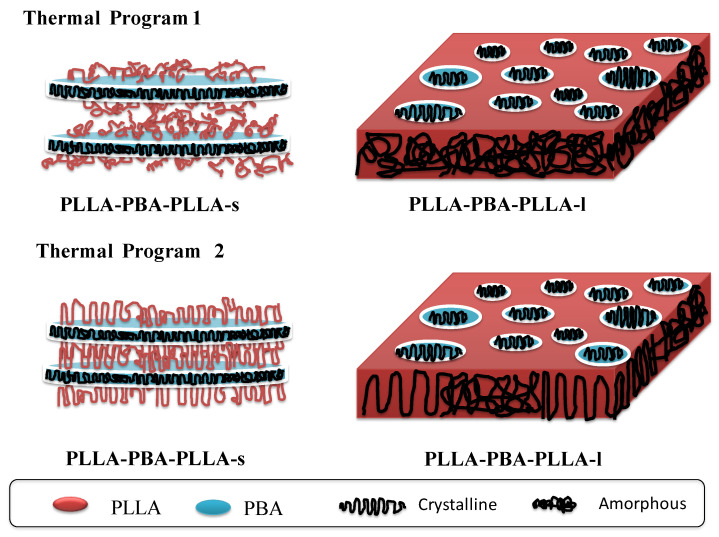
A scheme showing the resultant microstructures of PLLA-PBA-PLLA triblock copolymers under thermal programs 1 and 2, respectively.

According to the results of WAXD and DSC, it could be found that not only the f_PBA_ but also the state (crystallization or amorphous) of the PLLA blocks manipulate the polymorphic crystallization of PBA-mid within the PLLA-PBA-PLLA copolymers. Schmitt and Mahanthappa [32] concluded that when 52% ≤ f_PBA_ ≤ 75%, the PBA block within PLLA-PBA-PLLA copolymers formed a stable lamellar mesophase in melts. Meanwhile, they also confirmed that when f_PBA_ ≤ 40%, the PBA block within PLLA-PBA-PLLA copolymers might form cylindrical phases in melts. In this case, the f_PBA_ of PLLA-PBA-PLLA-s is about 68 % and could predict that PBA-mid might display an apparent formation of a lamellar domain structure in the melts. On the other hand, the f_PBA_ within PLLA-PBA-PLLA-l is about 35%, so PBA-mid might be located as nanoscaled cylindrical phases in the melts. Figure 8 shows the resultant microstructures of PLLA-PBA-PLLA copolymers under the thermal programs 1 and 2, respectively.

In the thermal program 1, when quenching from 200 °C to the T_c,PBA_ directly, the PLLA rigid amorphous phase coexists with the semi-crystallized PBA-mid lamellar domains for PLLA-PBA-PLLA-s. It can be concluded from this that the majority of chains of PBA-mid might crystallize within their nanoscaled lamellar domains, while the amorphous PLLA blocks might locate between the PBA-mid lamellar domains. Therefore, the segments of the major PBA-mid are restricted within their own nanoscaled lamellar domains, resulting in the crystallization temperatures of the α and β crystals shifting to the lower temperature region. Meanwhile, the nanoscaled cylindrical domains of PBA-mid are surrounded by the continuous PLLA amorphous phase for PLLA-PBA-PLLA-l. The mobility of PBA-mid segments for PLLA-PBA-PLLA-l is not only confined within these nanoscaled cylindrical domains but also hindered by linkages with the random coil region of the amorphous PLLA. Thus, the crystallization behavior of PBA-mid could not be observed.

In the thermal program 2, when PLLA-PBA-PLLA copolymers were quenched from the microphase-separated melt into low temperatures, the PLLA blocks crystallized first to form solid lamellar morphology, an alternating structure consisting of crystallized PLLA lamellae and amorphous PBA-mid phase, followed by the crystallization of PBA-mid starting from this morphology. After being cooled to ambient temperature, the chains of PBA-mid could crystallize within their nanoscaled lamellar domains for PLLA-PBA-PLLA-s. During the crystallization process, the segments of PBA-mid within PLLA-PBA-PLLA-s are restricted within nanoscaled lamellar domains, resulting in the crystallization temperatures of the α and β crystals shifting to the lower temperature region. However, it was difficult for us to identify the apparent formation of α and β crystals of the PBA-mid for PLLA-PBA-PLLA-l. The reason for this could be explained because the PBA-mid within the PLLA-PBA-PLLA-l might form the nanoscaled cylindrical domain surrounded by the PLLA continuous phase. In such a case, PLLA lamellar morphology will be a kind of spatial confinement against the subsequent crystallization of PBA-mid. This confinement is expected to be intermediate between hard confinement by glassy lamellar microdomains and soft confinement by rubbery ones, because the crystallized PLLA lamellae consist of PLLA crystals covered with amorphous PLLA blocks. During the crystallization process, some chains of PBA-mid were spatially confined within the nanoscaled cylindrical domains. Other chains of PBA-mid might locate themselves on the interface domains between the PLLA and PBA or arrange themselves within the PLLA interlamellar space, resulting in the phenomena of the polymorphic crystallization behavior of PBA-mid not being detected clearly.

### 3.4. Phase Transition of PBA-mid upon Annealing Analyzed by WAXD

**Figure 9:**
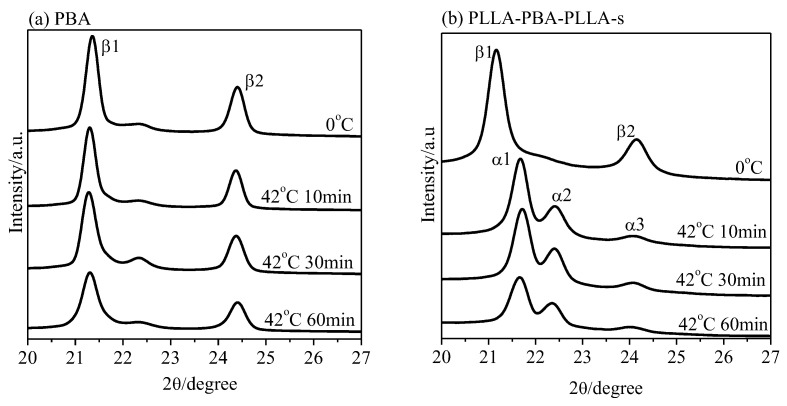
WAXD patterns of (**a**) PBA and (**b**) PLLA-PBA-PLLA-s after initial melt crystallization at 0 °C followed by annealing at 42 °C for different periods of time. (PLLA was held in its amorphous state).

**Figure 10:**
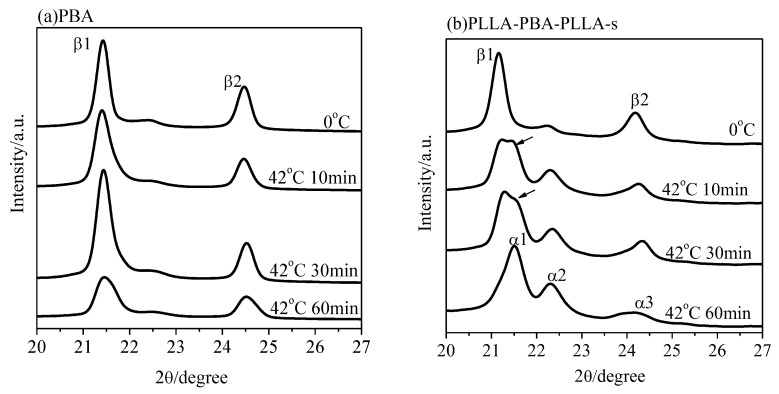
WAXD patterns of (**a**) PBA and (**b**) PLLA-PBA-PLLA-s after initial melt crystallization at 0 °C followed by annealing at 42 °C for different periods of time. (PLLA was held in its crystallized state).

The phase transition behavior of PBA-mid upon annealing was investigated by WAXD. The diffraction peaks of PBA-mid within PLLA-PBA-PLLA-l could not be detected clearly due to the lower volume fraction of PBA-mid (f_PBA_ = 35%). The crystallization behavior of PBA-mid within the PLLA-PBA-PLLA-s is shown in Figure 9 and Figure 10, respectively.

Figure 9 shows the WAXD patterns of PBA and PLLA-PBA-PLLA-s after being annealed at 42 °C for different periods of time after initially being crystallized at 0 °C to develop the pure β form, with the PLLA blocks being held in their amorphous state in this case. As shown in Figure 9a, after annealing at 42 °C for 10, 30, and 60 min, no diffraction peak related to the α-form crystal could be discerned for the neat PBA. However, for PLLA-PBA-PLLA-s, as shown in Figure 9b, the β-form crystal of PBA-mid is quickly and completely transformed into the α-form crystal within 10 min, suggesting that it is easy for the PBA-mid phase to transform from the metastable β-form crystal of PBA-mid to the stable α-form crystal within PLLA-PBA-PLLA-s when PLLA is in its amorphous state.

Figure 10 shows the WAXD patterns of PBA and PLLA-PBA-PLLA-s after being annealed at 42 °C for different periods of time after initially being crystallized at 0 °C to develop the pure β-form crystal when PLLA was held in its crystalline phase. The weak peak (pointed at by the arrows), related to the α-form crystal of PBA-mid within PLLA-PBA-PLLA-s, could be detected within 10 and 30 min, respectively. Furthermore, if the annealing time is raised to 60 min, the β-form crystal is completely transformed into the α-form crystal, as shown in Figure 10b. However, no obvious diffraction peaks related to the α-form crystal could be observed for PBA after annealing at 42 °C for 10, 30, and 60 min.

It has been reported that annealing PBA at 40 °C for 1 h only results in the thickening of the lamellae while the energy available for the phase transition for β-form crystals to α-form crystals is not enough [1]. This means that the accumulated energy after annealing at 40 °C for 1 h is still lower than the critical energy barrier used for phase transition [33]. The crystallization temperature of the α form of PBA-mid within PLLA-PBA-PLLA-s decreases, which suggests that the critical energy barrier for the phase transition decreases too. The greater temperature difference between the annealing temperature and crystallization temperature of PBA-mid and those of the neat PBA provide more energy to accelerate the phase transition. The energy obtained from annealing is the predominant driving force resulting in phase transformation. However, for PLLA-PBA-PLLA-s, the transforming rate of the β-form crystal to the stable α crystal under the thermal program 1 is faster than that of the thermal program 2. The reason might be that the segments of PBA-mid within PLLA-PBA-PLLA-s are restricted within nanoscaled lamellar domains during the annealing process under the thermal program 1. While in the thermal program 2, it seems a little complicated that the lamellar domains of PBA-mid coexist with the crystal and amorphous phases of PLLA. It is predicted that the mobility of PBA-mid chains is not only restricted within nanoscaled lamellar domains but also by the crystalline and amorphous states of PLLA, which are unfavorable for the adjustment of PBA-mid chains during the annealing process, resulting in the lower transforming rate of the β-form crystal to the stable α form.

### 3.5. Spherulitic Morphology Observation by POM

**Figure 11:**
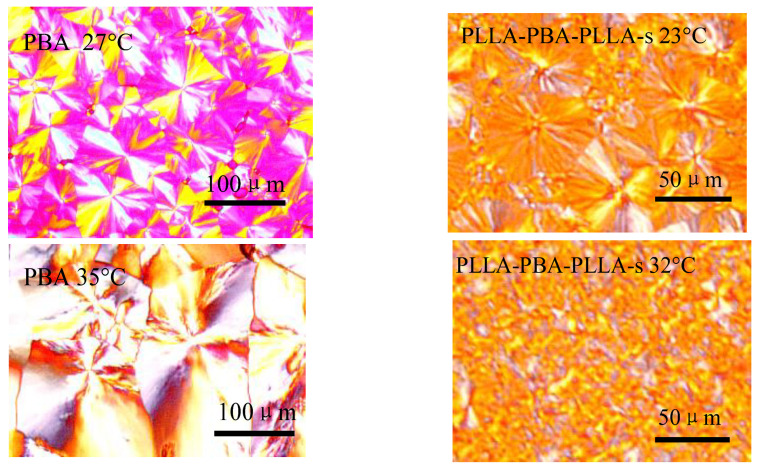
POM micrographs of PBA and PLLA-PBA-PLLA-s following melt crystallization at the characteristic temperatures under the thermal program 1.

Figure 11 shows the POM micrographs of PBA and PLLA-PBA-PLLA-s following melt crystallization at various temperatures under the thermal program 1. The POM photos were taken at the characteristic temperatures of the two samples, at which α, β crystal types appear. Eventually, the POM spherulite morphologies of the neat PBA well agreed with the results of the DSC and WAXD analyses above. However, the segregated domains are a lot smaller and finer; thus, it was difficult for us to clearly identify them at the given temperatures. The brightness that is attributed to the refringence of the large amount of PBA spherulite is much stronger, suggesting that the predominant region is occupied by PBA spherulites and PLLA is only sporadically dispersed among the PBA spherulites. Furthermore, it can be observed that the spherulite morphologies of PBA-mid within the copolymers correspond to the α crystal form of neat PBA, at T_c,PBA_ = 32 °C. The POM photos provide a direct image and evidence of the polymorphic crystals of PBA-mid within PLLA-PBA-PLLA copolymers, which were confirmed by the WAXD and DSC analyses discussed above.

## 4. Discussion

By comparing the results of WAXD and DSC under the two thermal programs, it is now clear that the polymorphic crystallization behavior of PBA-mid within PLLA-PBA-PLLA triblock copolymers depends on the state (crystallization or amorphous) of the PLLA component and f_PBA_ within the PLLA-PBA-PLLA. In the thermal program 1, the majority of chains of PBA-mid might crystallize within their lamellar nanoscaled domains, resulting in the crystallization temperatures of the α and β crystals shifting to the lower temperature region. PBA-mid for PLLA-PBA-PLLA-l is not only confined within nanoscaled cylindrical domains, but also hindered by linkage with the random coil region of amorphous PLLA, resulting in the crystallization behavior of PBA-mid not being able to be observed. In the thermal program 2, during the crystallization process, the segments of PBA-mid chains within PLLA-PBA-PLLA-s are restricted within nanoscaled lamellar domains, resulting in the crystallization temperatures of the α and β crystals shifting to the lower temperature region too. However, the PBA-mid within PLLA-PBA-PLLA-l might crystallize within the cylindrical domains, or locate itself on the interface domain between PLLA and PBA, or even arrange within the PLLA interlamellar space, which results in the phenomena of polymorphic crystallization behavior of PBA-mid not being clearly detectable.

Furthermore, the phase transition behavior of PBA-mid within PLLA-PBA-PLLA-s upon annealing could be confirmed by WAXD. It was also interesting to find that the α-type crystal of PBA-mid within PLLA-PBA-PLLA-s was favorable to develop in the lower temperature region regardless of the state (crystallization or amorphous) of the PLLA component, which means that a large proportion of PBA-mid chains within PLLA-PBA-PLLA-s is restricted and crystallized within nanoscaled lamellar domains. Thus, the state (crystallization or amorphous) of the PLLA component has little effect on the crystallization behavior of PBA-mid.

Additionally, when the PLLA component was held in its amorphous state, it was easier for PBA-mid within the PLLA-PBA-PLLA-s to transform from the metastable β-form crystal to the stable α-form crystal. This means that the crystallized PLLA lamellae consisting of PLLA crystals covered with amorphous PLLA blocks are a kind of spatial confinement against the subsequent crystallization of PBA-mid.

## 5. Conclusions

In conclusion, new, biodegradable, aliphatic PLLA-PBA-PLLA triblock copolymers with soft PBA and hard PLLA building blocks were synthesized via ROP successfully. It could be found that not only did the f_PBA_ of the PLLA-PBA-PLLA copolymers, but also, the state (crystallization or amorphous) of the PLLA blocks manipulate the polymorphic crystallization of PBA-mid within the PLLA-PBA-PLLA copolymers. On the basis of the f_PBA_ within the copolymer, it was predicted that PBA-mid within PLLA-PBA-PLLA-s might display lamellar domain structure. On the other hand, PBA-mid within PLLA-PBA-PLLA-l might locate itself in nanoscaled cylindrical domains surrounded by PLLA continuous phase. It was interesting to find that the α-type crystal of PBA-mid is favorable to develop at a lower temperature regardless of the condition (amorphous or crystallization) of the PLLA component. Additionally, it is easier for PBA-mid phase within PLLA-PBA-PLLA copolymers to transform from the metastable β-form crystal to the stable α-form crystal, when the PLLA component is held in its amorphous state rather than in its crystalline state. Furthermore, POM photos might provide direct evidence of the polymorphic crystallization of PBA-mid within PLLA-PBA-PLLA-s.

It is hoped that this investigation of the polymorphic crystallization behavior of ABA triblock copolymers could establish a model system to regulate the structure of polymorphic polymers in semicrystalline/semicrystalline copolymers. These results might be beneficial for the investigation of the crystallization behavior related to the mechanical and physical properties of ABA triblock copolymers, which would be applied in the potential industrial areas.

## Figures and Tables

**Table 1:** Volume fraction, molecular weight, crystallization temperature, and melting temperature of PBA and PLLA, and within PLLA-PBA-PLLA copolymers.

*Sample*	*f_PBA_^a^*	*Mn _GPC_*	*Mw _GPC_*	*Mw/Mn _GPC_*	*Mn _NMR_*	*T_c_^e^*		*Tm ^f^*	
	*%*	*(g/mol)*	*(g/mol)*			*/°C*		*/°C*	
PBA	100	8219	13,235	1.61	7920	31.9		55.6	
PLLA-PBA-PLLA-s ^b^	68	13,479	23,511	1.74	13,200	31.1	76	50	137
PLLA-PBA-PLLA-l ^c^	35	29,818	54.073	1.81	28,512	-	90	-	160
PLLA ^d^	0	25,052	48,625	1.89	-		103		167

a: f_PBA_ is the volume fraction PBA-mid in the PLLA-PBA-PLLA copolymers was estimated from the result of ^1^HNMR and corrected by density. b: PLLA-PBA-PLLA-s, PLLA-PBA-PLLA triblock copolymer with a short PLLA block length. c: PLLA-PBA-PLLA-l, PLLA-PBA-PLLA triblock copolymer with a long PLLA block length. d: The PLLA (Mn = 20,000) applied in this research was prepared by ring-opening polymerization. e: T_c,PBA_ and T_c,PLLA_ are the crystallization temperatures of the PBA-mid and PLLA blocks within the copolymers, respectively. f: T_m,PBA_ and T_m,PLLA_ are indicative of the melting temperatures of the PBA-mid and PLLA blocks within the copolymers, respectively.

**Table 2:** Melt crystallization temperature of α, β, and α + β crystal forms of samples summarized from DSC and WAXD.

Sample	Instrument	Thermal Program 1			Thermal Program 2		
		α	α + β	β	α	α + β	β
		/°C	/°C	/°C	/°C	/°C	/°C
PBA	DSC	34	33~31	30	35	34~30	29
	WAXD	34	33~30	29	34	33~30	29
PLLA-PBA-PLLA-s	DSC	30	29~26	25	30	29~25	24
	WAXD	30	29~24	23	29	28~25	24
PLLA-PBA-PLLA-l	DSC	0	−	−	31	30~28	27
	WAXD	−	−	−	+	+	+

+: able to be detected, but unclear; −: unable to be detected.

## Data Availability

Not applicable.
